# Supplementary figures and images for: Establishment of a Prognostic Prediction and Drug Selection Model for Patients with Clear Cell Renal Cell Carcinoma by Multiomics Data Analysis
Source: Oxid Med Cell Longev. 2022 Jan 4;2022:3617775. doi: 10.1155/2022/3617775 (PMC8752262; doi:10.1155/2022/3617775)

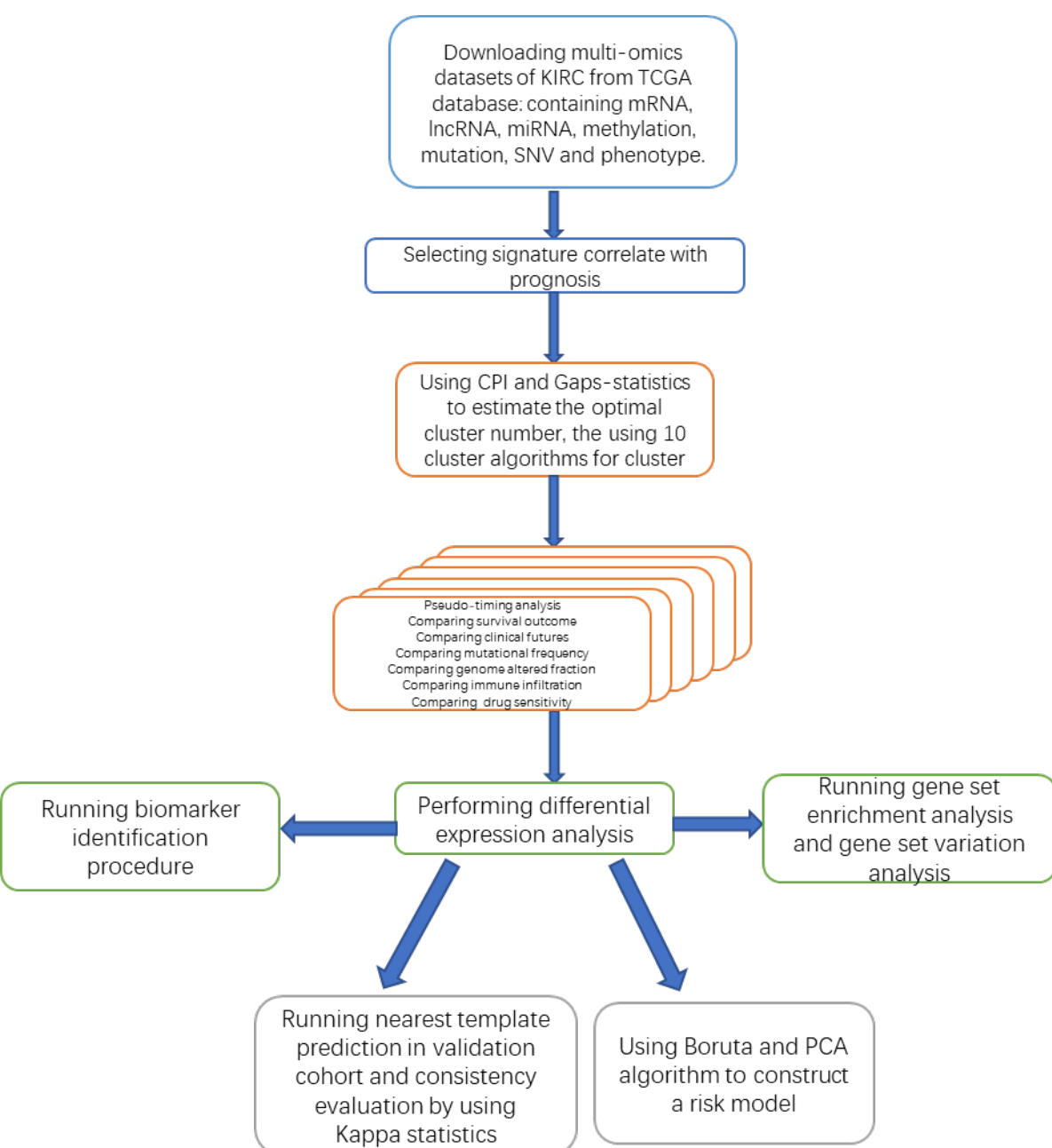

**Figure S1**

Supplement: Supplementary Materials — Figure S1: the workflow of this study. Figure S2: (A) identification of the optimal cluster number by calculating CPI (blue line) and Gaps-statistics (red line) in TCGA-KIRC cohort. (B) Consensus heatmap based on results from 10 multiomics integrative clustering algorithms with the cluster number of 2, 3, 4, and 5, and related quantification of sample similarity using the Silhoutte score based on the consensus ensembles result. Figure S3: heatmap of subtype-specific upregulated and downregulated biomarkers using DEseq2 for the two identified subtypes in TCGA-KIRC cohort. Figure S4: (A) DNA substitution types including transition (Ti) and transversion (Tv) among CS1 and CS2. (B) The most significant difference of mutated genes between CS1 and CS2. (C) The lollipop plot illustrates the differential distribution of variants for PBRM1. (D) Kaplan-Meier curves show the independent relevance between overall survival and PBRM1 mutation in CS1 and CS2 subgroups. (E) The lollipop plot illustrates the differential distribution of variants for PBRM1. (F) Potential druggable gene categories from mutation dataset in CS1 and CS2. Figure S5: (A) differences in oncogenic signaling pathways between the CS1 and CS2 subgroups. (B) Heatmap of oncogenic pathways based on ssGSEA algorithm in the CS1 and CS2 subgroups. Figure S6: heatmap of oncogenic pathways based on the ssGSEA algorithm in the CS1 and CS2 subgroups. Figure S7: (A) heatmap of the immune-related gene family of chemokines and receptors, coinhibitors, costimulators, interferons, and receptors and MHC expression in the CS1 and CS2 subgroups. (B) Expression of immune checkpoints in CS1 and CS2 subgroups and difference in ESTIMATE scores between the subgroups. Figure S8: the structure tomographs of the candidate small-molecule drugs for the CS1 subgroup. Figure S9: (A) heatmap of NTP in outer KIRC cohort using subtype-specific upregulated biomarkers identified from Japan ccRCC cohort. (B) Kaplan-Meier survival curve of the two [file 3617775.f1.zip › FigureS1 (1).pdf]

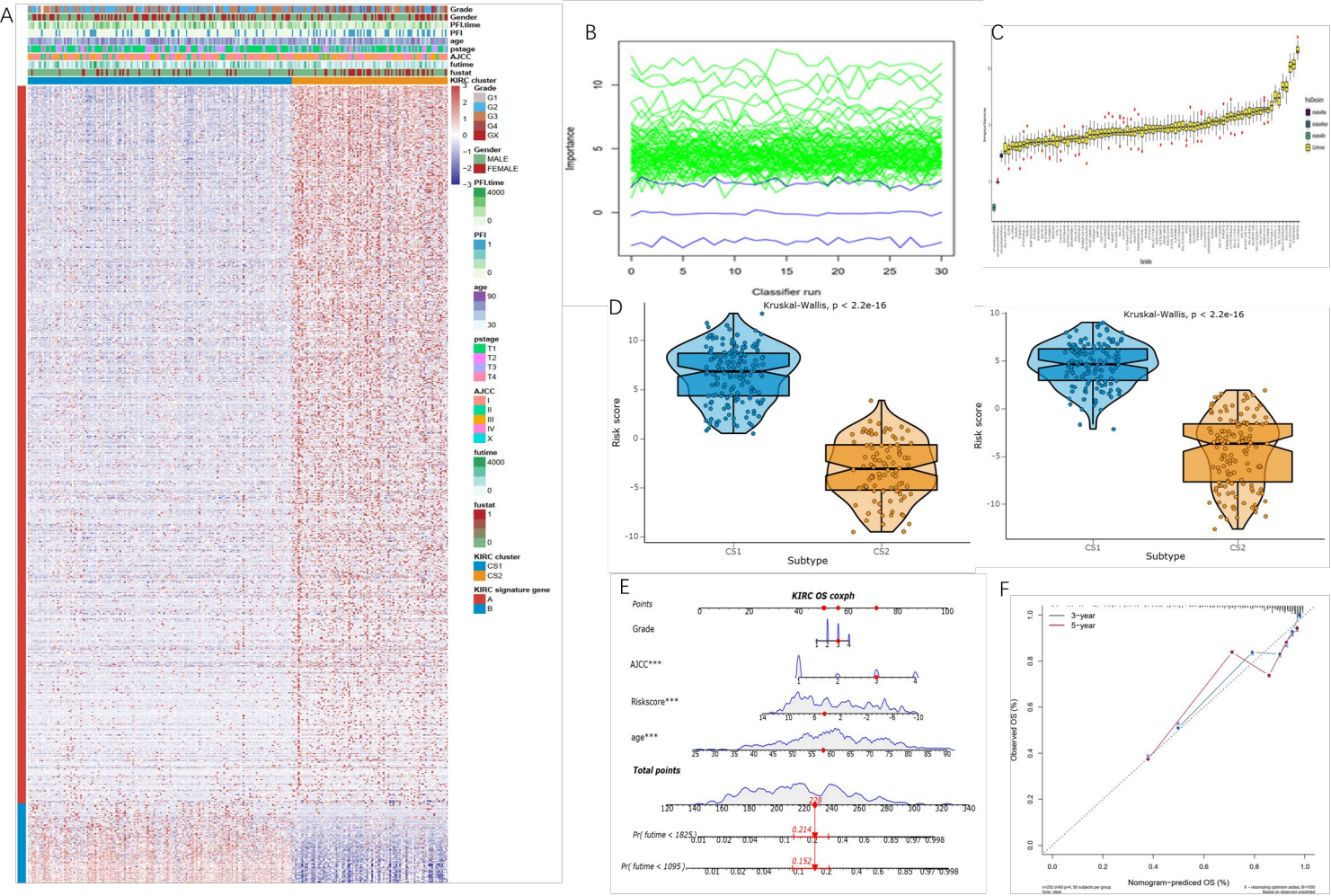

Figure S10

Supplement: Supplementary Materials — Figure S1: the workflow of this study. Figure S2: (A) identification of the optimal cluster number by calculating CPI (blue line) and Gaps-statistics (red line) in TCGA-KIRC cohort. (B) Consensus heatmap based on results from 10 multiomics integrative clustering algorithms with the cluster number of 2, 3, 4, and 5, and related quantification of sample similarity using the Silhoutte score based on the consensus ensembles result. Figure S3: heatmap of subtype-specific upregulated and downregulated biomarkers using DEseq2 for the two identified subtypes in TCGA-KIRC cohort. Figure S4: (A) DNA substitution types including transition (Ti) and transversion (Tv) among CS1 and CS2. (B) The most significant difference of mutated genes between CS1 and CS2. (C) The lollipop plot illustrates the differential distribution of variants for PBRM1. (D) Kaplan-Meier curves show the independent relevance between overall survival and PBRM1 mutation in CS1 and CS2 subgroups. (E) The lollipop plot illustrates the differential distribution of variants for PBRM1. (F) Potential druggable gene categories from mutation dataset in CS1 and CS2. Figure S5: (A) differences in oncogenic signaling pathways between the CS1 and CS2 subgroups. (B) Heatmap of oncogenic pathways based on ssGSEA algorithm in the CS1 and CS2 subgroups. Figure S6: heatmap of oncogenic pathways based on the ssGSEA algorithm in the CS1 and CS2 subgroups. Figure S7: (A) heatmap of the immune-related gene family of chemokines and receptors, coinhibitors, costimulators, interferons, and receptors and MHC expression in the CS1 and CS2 subgroups. (B) Expression of immune checkpoints in CS1 and CS2 subgroups and difference in ESTIMATE scores between the subgroups. Figure S8: the structure tomographs of the candidate small-molecule drugs for the CS1 subgroup. Figure S9: (A) heatmap of NTP in outer KIRC cohort using subtype-specific upregulated biomarkers identified from Japan ccRCC cohort. (B) Kaplan-Meier survival curve of the two [file 3617775.f1.zip › FigureS10 (1).pdf]

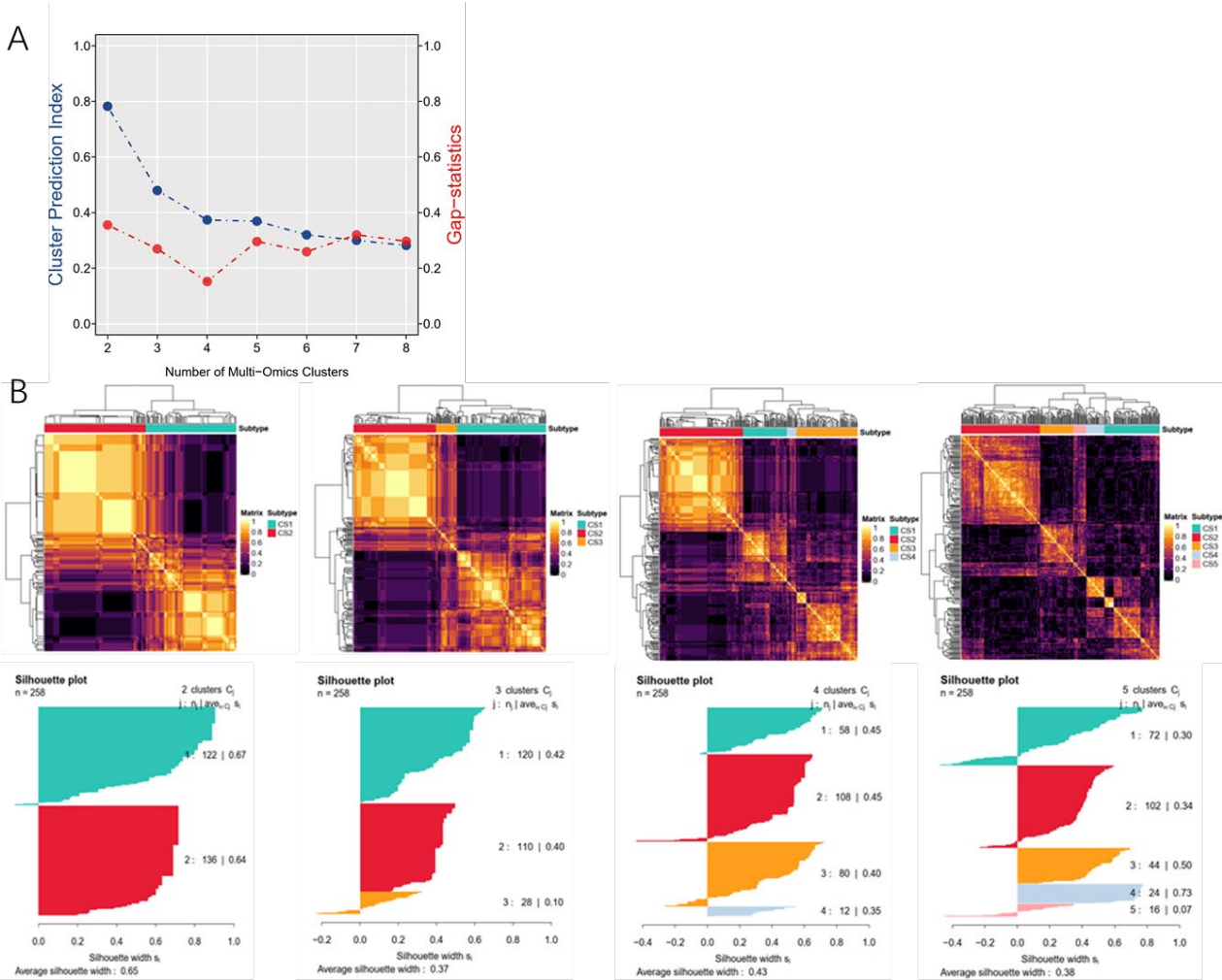

Figure S2

Supplement: Supplementary Materials — Figure S1: the workflow of this study. Figure S2: (A) identification of the optimal cluster number by calculating CPI (blue line) and Gaps-statistics (red line) in TCGA-KIRC cohort. (B) Consensus heatmap based on results from 10 multiomics integrative clustering algorithms with the cluster number of 2, 3, 4, and 5, and related quantification of sample similarity using the Silhoutte score based on the consensus ensembles result. Figure S3: heatmap of subtype-specific upregulated and downregulated biomarkers using DEseq2 for the two identified subtypes in TCGA-KIRC cohort. Figure S4: (A) DNA substitution types including transition (Ti) and transversion (Tv) among CS1 and CS2. (B) The most significant difference of mutated genes between CS1 and CS2. (C) The lollipop plot illustrates the differential distribution of variants for PBRM1. (D) Kaplan-Meier curves show the independent relevance between overall survival and PBRM1 mutation in CS1 and CS2 subgroups. (E) The lollipop plot illustrates the differential distribution of variants for PBRM1. (F) Potential druggable gene categories from mutation dataset in CS1 and CS2. Figure S5: (A) differences in oncogenic signaling pathways between the CS1 and CS2 subgroups. (B) Heatmap of oncogenic pathways based on ssGSEA algorithm in the CS1 and CS2 subgroups. Figure S6: heatmap of oncogenic pathways based on the ssGSEA algorithm in the CS1 and CS2 subgroups. Figure S7: (A) heatmap of the immune-related gene family of chemokines and receptors, coinhibitors, costimulators, interferons, and receptors and MHC expression in the CS1 and CS2 subgroups. (B) Expression of immune checkpoints in CS1 and CS2 subgroups and difference in ESTIMATE scores between the subgroups. Figure S8: the structure tomographs of the candidate small-molecule drugs for the CS1 subgroup. Figure S9: (A) heatmap of NTP in outer KIRC cohort using subtype-specific upregulated biomarkers identified from Japan ccRCC cohort. (B) Kaplan-Meier survival curve of the two [file 3617775.f1.zip › FigureS2 (1).pdf]

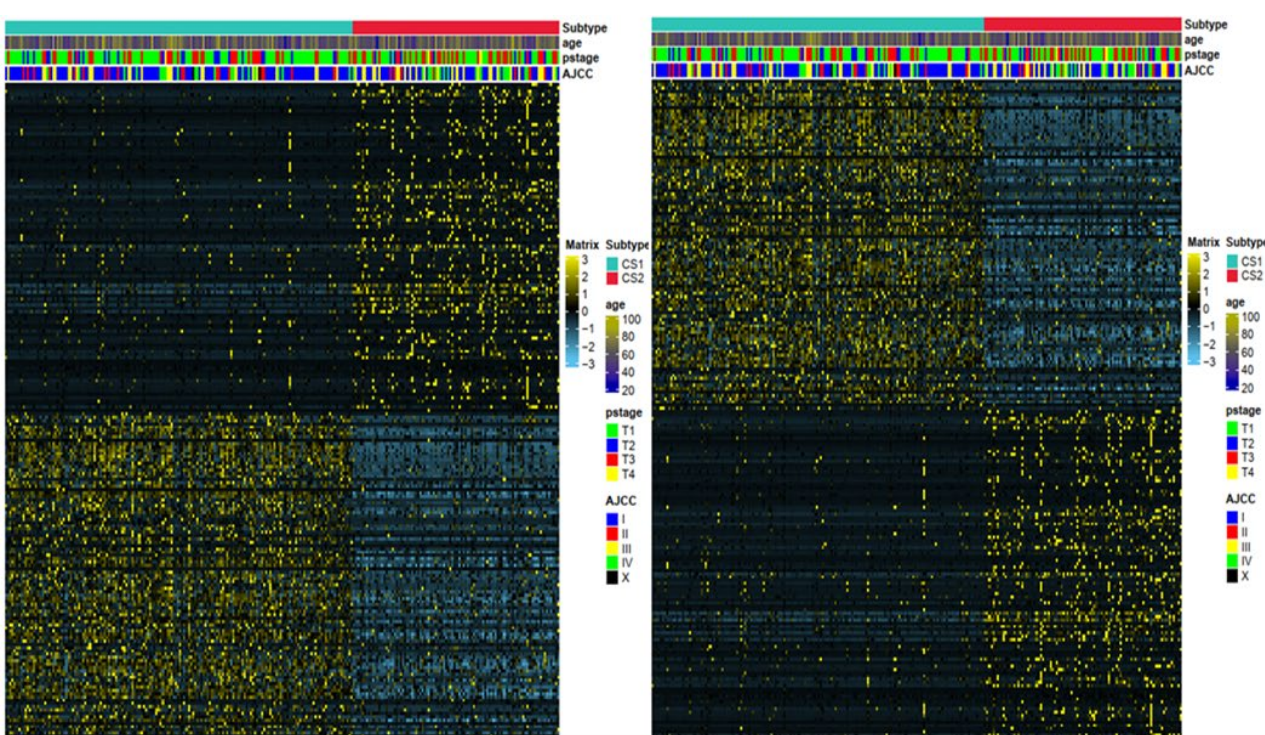

Figure S3

Supplement: Supplementary Materials — Figure S1: the workflow of this study. Figure S2: (A) identification of the optimal cluster number by calculating CPI (blue line) and Gaps-statistics (red line) in TCGA-KIRC cohort. (B) Consensus heatmap based on results from 10 multiomics integrative clustering algorithms with the cluster number of 2, 3, 4, and 5, and related quantification of sample similarity using the Silhoutte score based on the consensus ensembles result. Figure S3: heatmap of subtype-specific upregulated and downregulated biomarkers using DEseq2 for the two identified subtypes in TCGA-KIRC cohort. Figure S4: (A) DNA substitution types including transition (Ti) and transversion (Tv) among CS1 and CS2. (B) The most significant difference of mutated genes between CS1 and CS2. (C) The lollipop plot illustrates the differential distribution of variants for PBRM1. (D) Kaplan-Meier curves show the independent relevance between overall survival and PBRM1 mutation in CS1 and CS2 subgroups. (E) The lollipop plot illustrates the differential distribution of variants for PBRM1. (F) Potential druggable gene categories from mutation dataset in CS1 and CS2. Figure S5: (A) differences in oncogenic signaling pathways between the CS1 and CS2 subgroups. (B) Heatmap of oncogenic pathways based on ssGSEA algorithm in the CS1 and CS2 subgroups. Figure S6: heatmap of oncogenic pathways based on the ssGSEA algorithm in the CS1 and CS2 subgroups. Figure S7: (A) heatmap of the immune-related gene family of chemokines and receptors, coinhibitors, costimulators, interferons, and receptors and MHC expression in the CS1 and CS2 subgroups. (B) Expression of immune checkpoints in CS1 and CS2 subgroups and difference in ESTIMATE scores between the subgroups. Figure S8: the structure tomographs of the candidate small-molecule drugs for the CS1 subgroup. Figure S9: (A) heatmap of NTP in outer KIRC cohort using subtype-specific upregulated biomarkers identified from Japan ccRCC cohort. (B) Kaplan-Meier survival curve of the two [file 3617775.f1.zip › FigureS3 (1).pdf]

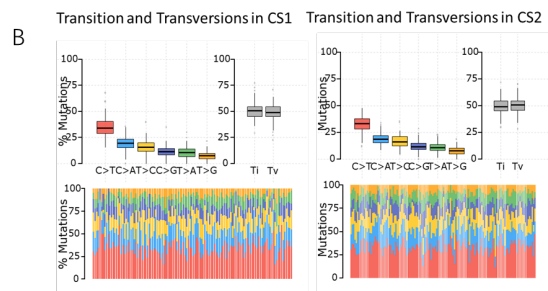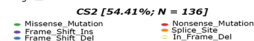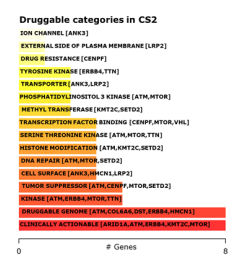

**Figure S4**

Supplement: Supplementary Materials — Figure S1: the workflow of this study. Figure S2: (A) identification of the optimal cluster number by calculating CPI (blue line) and Gaps-statistics (red line) in TCGA-KIRC cohort. (B) Consensus heatmap based on results from 10 multiomics integrative clustering algorithms with the cluster number of 2, 3, 4, and 5, and related quantification of sample similarity using the Silhoutte score based on the consensus ensembles result. Figure S3: heatmap of subtype-specific upregulated and downregulated biomarkers using DEseq2 for the two identified subtypes in TCGA-KIRC cohort. Figure S4: (A) DNA substitution types including transition (Ti) and transversion (Tv) among CS1 and CS2. (B) The most significant difference of mutated genes between CS1 and CS2. (C) The lollipop plot illustrates the differential distribution of variants for PBRM1. (D) Kaplan-Meier curves show the independent relevance between overall survival and PBRM1 mutation in CS1 and CS2 subgroups. (E) The lollipop plot illustrates the differential distribution of variants for PBRM1. (F) Potential druggable gene categories from mutation dataset in CS1 and CS2. Figure S5: (A) differences in oncogenic signaling pathways between the CS1 and CS2 subgroups. (B) Heatmap of oncogenic pathways based on ssGSEA algorithm in the CS1 and CS2 subgroups. Figure S6: heatmap of oncogenic pathways based on the ssGSEA algorithm in the CS1 and CS2 subgroups. Figure S7: (A) heatmap of the immune-related gene family of chemokines and receptors, coinhibitors, costimulators, interferons, and receptors and MHC expression in the CS1 and CS2 subgroups. (B) Expression of immune checkpoints in CS1 and CS2 subgroups and difference in ESTIMATE scores between the subgroups. Figure S8: the structure tomographs of the candidate small-molecule drugs for the CS1 subgroup. Figure S9: (A) heatmap of NTP in outer KIRC cohort using subtype-specific upregulated biomarkers identified from Japan ccRCC cohort. (B) Kaplan-Meier survival curve of the two [file 3617775.f1.zip › FigureS4 (1).pdf]

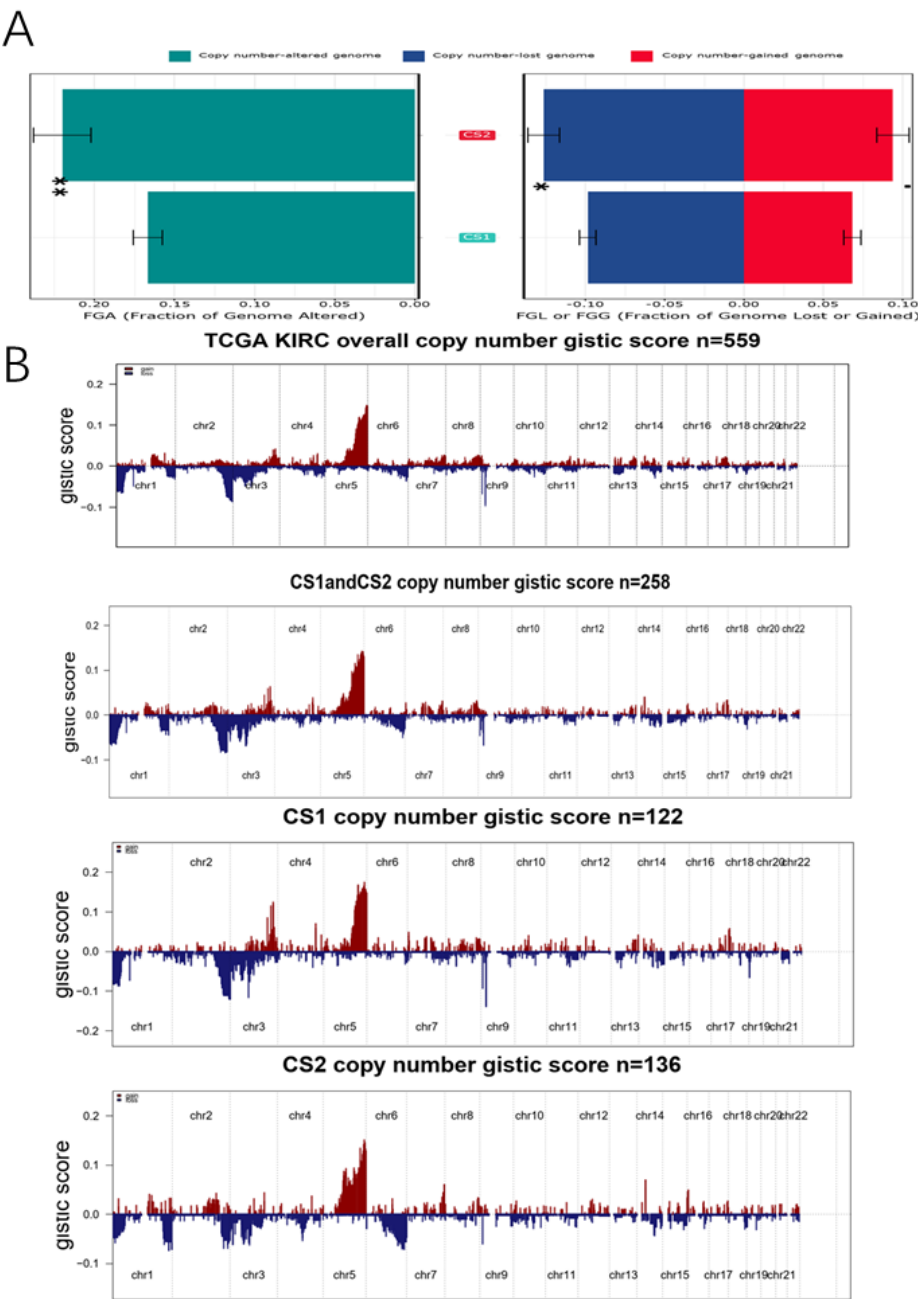

FigureS5

Supplement: Supplementary Materials — Figure S1: the workflow of this study. Figure S2: (A) identification of the optimal cluster number by calculating CPI (blue line) and Gaps-statistics (red line) in TCGA-KIRC cohort. (B) Consensus heatmap based on results from 10 multiomics integrative clustering algorithms with the cluster number of 2, 3, 4, and 5, and related quantification of sample similarity using the Silhoutte score based on the consensus ensembles result. Figure S3: heatmap of subtype-specific upregulated and downregulated biomarkers using DEseq2 for the two identified subtypes in TCGA-KIRC cohort. Figure S4: (A) DNA substitution types including transition (Ti) and transversion (Tv) among CS1 and CS2. (B) The most significant difference of mutated genes between CS1 and CS2. (C) The lollipop plot illustrates the differential distribution of variants for PBRM1. (D) Kaplan-Meier curves show the independent relevance between overall survival and PBRM1 mutation in CS1 and CS2 subgroups. (E) The lollipop plot illustrates the differential distribution of variants for PBRM1. (F) Potential druggable gene categories from mutation dataset in CS1 and CS2. Figure S5: (A) differences in oncogenic signaling pathways between the CS1 and CS2 subgroups. (B) Heatmap of oncogenic pathways based on ssGSEA algorithm in the CS1 and CS2 subgroups. Figure S6: heatmap of oncogenic pathways based on the ssGSEA algorithm in the CS1 and CS2 subgroups. Figure S7: (A) heatmap of the immune-related gene family of chemokines and receptors, coinhibitors, costimulators, interferons, and receptors and MHC expression in the CS1 and CS2 subgroups. (B) Expression of immune checkpoints in CS1 and CS2 subgroups and difference in ESTIMATE scores between the subgroups. Figure S8: the structure tomographs of the candidate small-molecule drugs for the CS1 subgroup. Figure S9: (A) heatmap of NTP in outer KIRC cohort using subtype-specific upregulated biomarkers identified from Japan ccRCC cohort. (B) Kaplan-Meier survival curve of the two [file 3617775.f1.zip › FigureS5 (1).pdf]

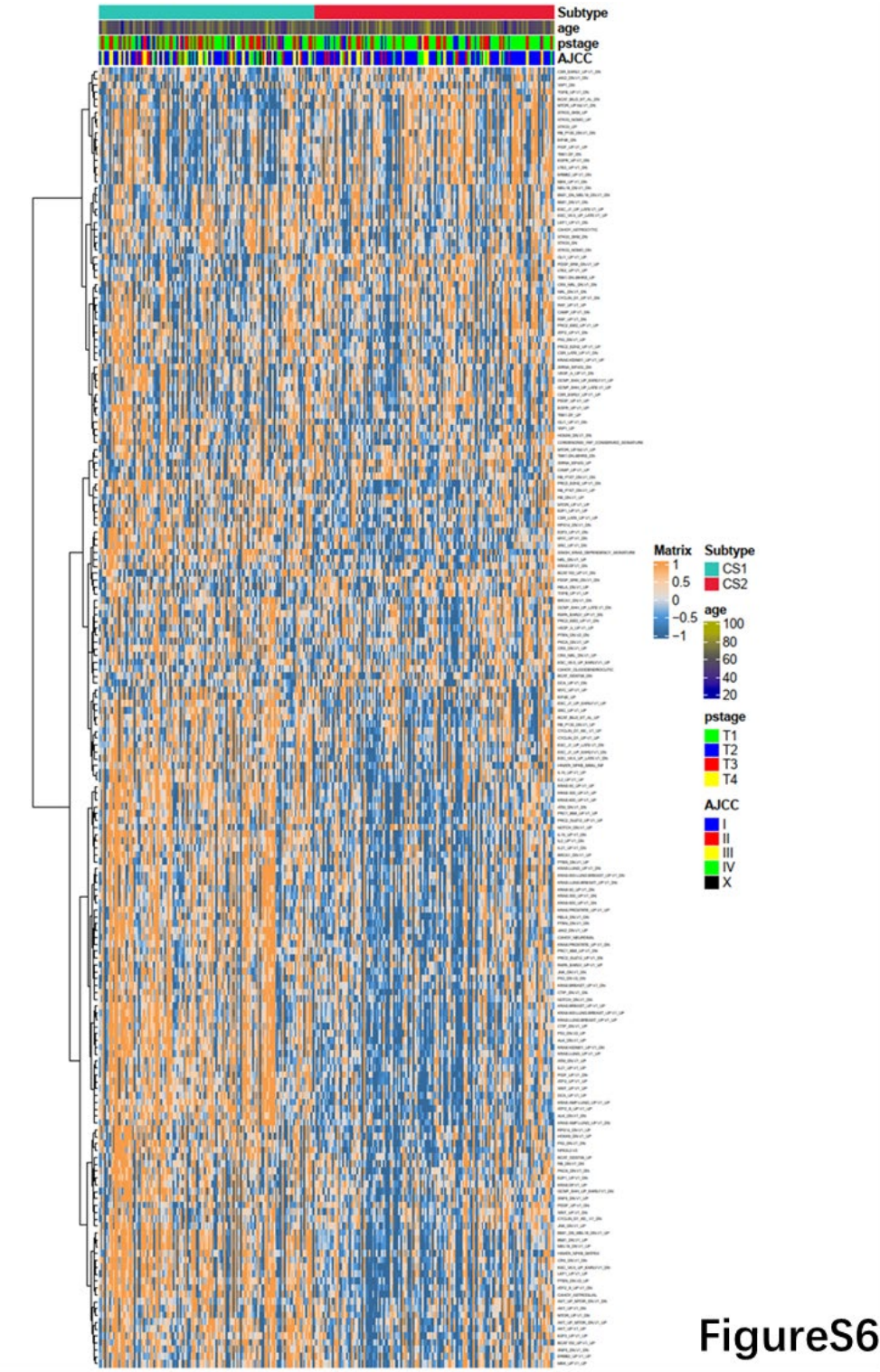

FigureS6

Supplement: Supplementary Materials — Figure S1: the workflow of this study. Figure S2: (A) identification of the optimal cluster number by calculating CPI (blue line) and Gaps-statistics (red line) in TCGA-KIRC cohort. (B) Consensus heatmap based on results from 10 multiomics integrative clustering algorithms with the cluster number of 2, 3, 4, and 5, and related quantification of sample similarity using the Silhoutte score based on the consensus ensembles result. Figure S3: heatmap of subtype-specific upregulated and downregulated biomarkers using DEseq2 for the two identified subtypes in TCGA-KIRC cohort. Figure S4: (A) DNA substitution types including transition (Ti) and transversion (Tv) among CS1 and CS2. (B) The most significant difference of mutated genes between CS1 and CS2. (C) The lollipop plot illustrates the differential distribution of variants for PBRM1. (D) Kaplan-Meier curves show the independent relevance between overall survival and PBRM1 mutation in CS1 and CS2 subgroups. (E) The lollipop plot illustrates the differential distribution of variants for PBRM1. (F) Potential druggable gene categories from mutation dataset in CS1 and CS2. Figure S5: (A) differences in oncogenic signaling pathways between the CS1 and CS2 subgroups. (B) Heatmap of oncogenic pathways based on ssGSEA algorithm in the CS1 and CS2 subgroups. Figure S6: heatmap of oncogenic pathways based on the ssGSEA algorithm in the CS1 and CS2 subgroups. Figure S7: (A) heatmap of the immune-related gene family of chemokines and receptors, coinhibitors, costimulators, interferons, and receptors and MHC expression in the CS1 and CS2 subgroups. (B) Expression of immune checkpoints in CS1 and CS2 subgroups and difference in ESTIMATE scores between the subgroups. Figure S8: the structure tomographs of the candidate small-molecule drugs for the CS1 subgroup. Figure S9: (A) heatmap of NTP in outer KIRC cohort using subtype-specific upregulated biomarkers identified from Japan ccRCC cohort. (B) Kaplan-Meier survival curve of the two [file 3617775.f1.zip › FigureS6 (1).pdf]

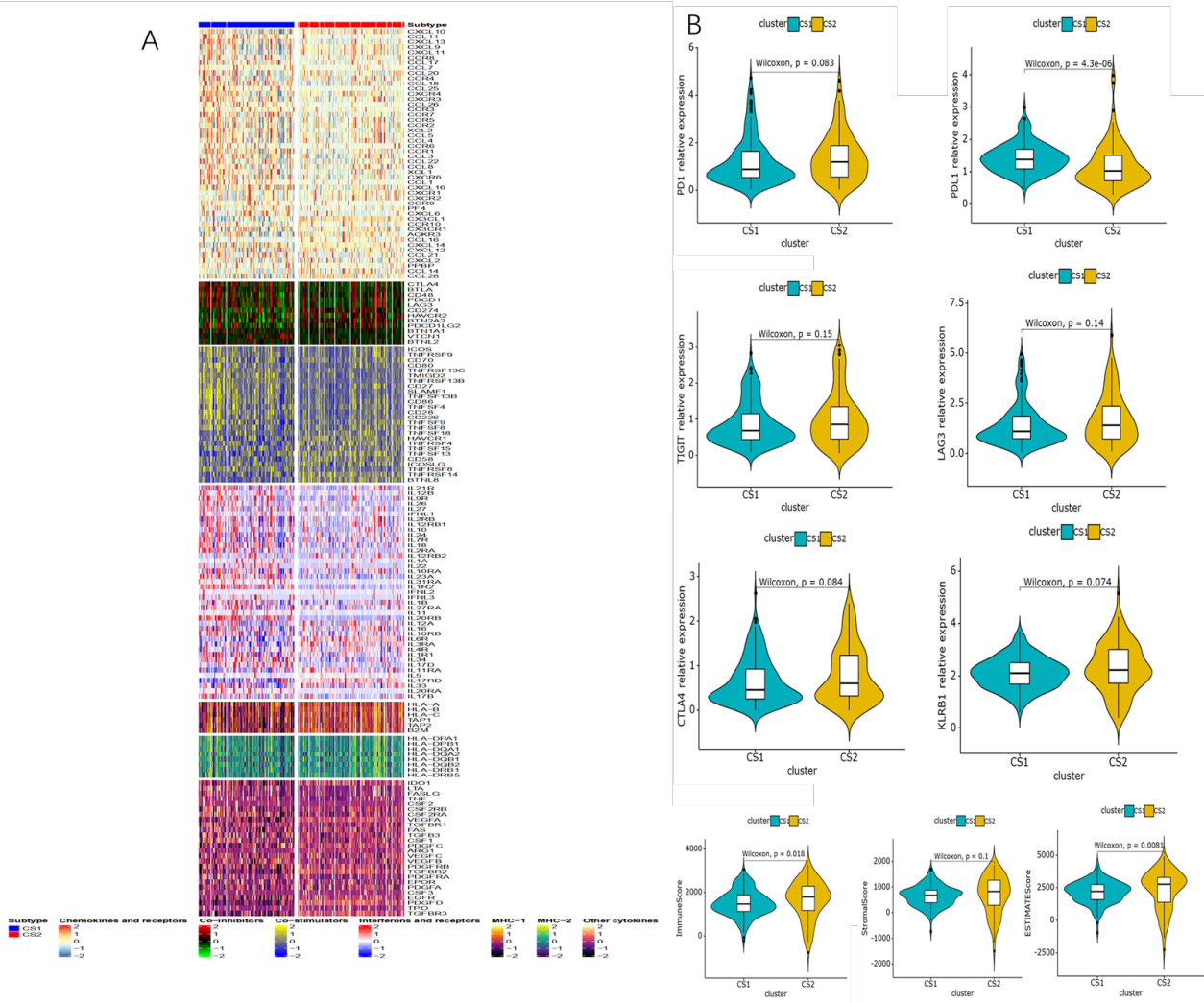

FigureS7

Supplement: Supplementary Materials — Figure S1: the workflow of this study. Figure S2: (A) identification of the optimal cluster number by calculating CPI (blue line) and Gaps-statistics (red line) in TCGA-KIRC cohort. (B) Consensus heatmap based on results from 10 multiomics integrative clustering algorithms with the cluster number of 2, 3, 4, and 5, and related quantification of sample similarity using the Silhoutte score based on the consensus ensembles result. Figure S3: heatmap of subtype-specific upregulated and downregulated biomarkers using DEseq2 for the two identified subtypes in TCGA-KIRC cohort. Figure S4: (A) DNA substitution types including transition (Ti) and transversion (Tv) among CS1 and CS2. (B) The most significant difference of mutated genes between CS1 and CS2. (C) The lollipop plot illustrates the differential distribution of variants for PBRM1. (D) Kaplan-Meier curves show the independent relevance between overall survival and PBRM1 mutation in CS1 and CS2 subgroups. (E) The lollipop plot illustrates the differential distribution of variants for PBRM1. (F) Potential druggable gene categories from mutation dataset in CS1 and CS2. Figure S5: (A) differences in oncogenic signaling pathways between the CS1 and CS2 subgroups. (B) Heatmap of oncogenic pathways based on ssGSEA algorithm in the CS1 and CS2 subgroups. Figure S6: heatmap of oncogenic pathways based on the ssGSEA algorithm in the CS1 and CS2 subgroups. Figure S7: (A) heatmap of the immune-related gene family of chemokines and receptors, coinhibitors, costimulators, interferons, and receptors and MHC expression in the CS1 and CS2 subgroups. (B) Expression of immune checkpoints in CS1 and CS2 subgroups and difference in ESTIMATE scores between the subgroups. Figure S8: the structure tomographs of the candidate small-molecule drugs for the CS1 subgroup. Figure S9: (A) heatmap of NTP in outer KIRC cohort using subtype-specific upregulated biomarkers identified from Japan ccRCC cohort. (B) Kaplan-Meier survival curve of the two [file 3617775.f1.zip › FigureS7 (1).pdf]

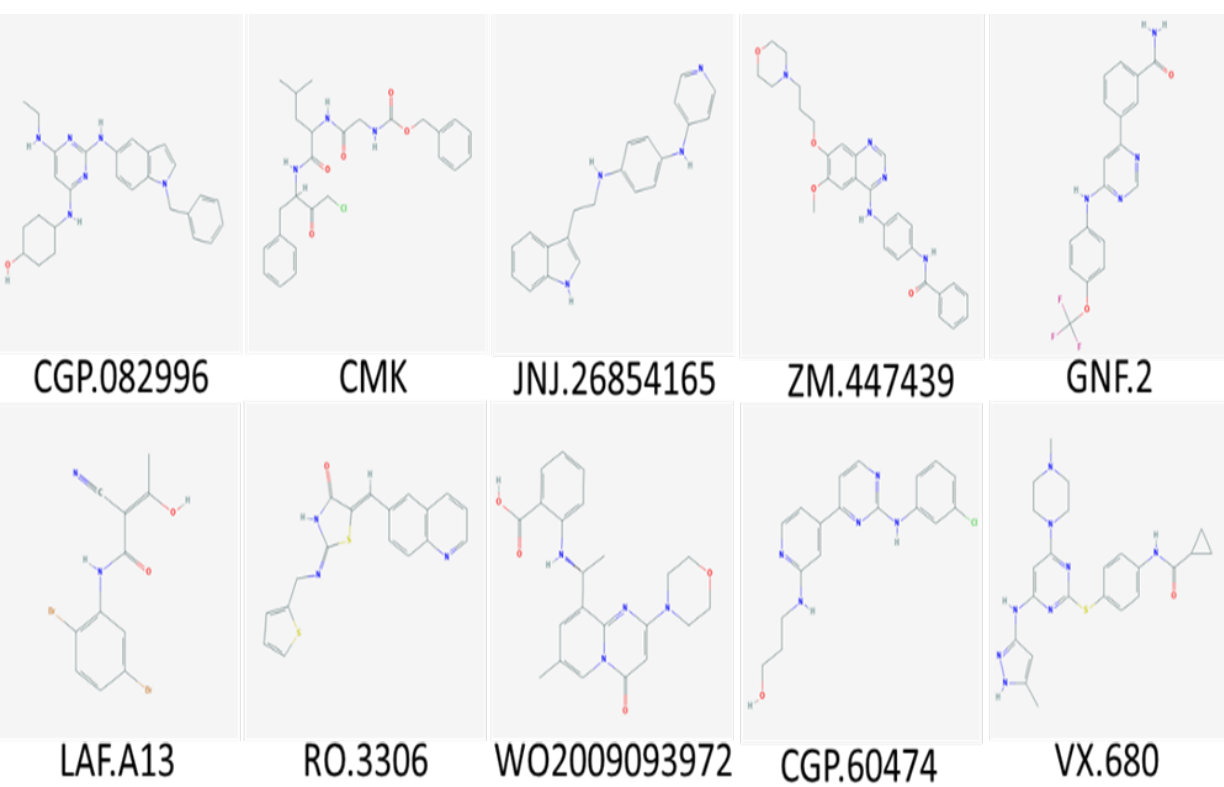

**Figure S8**

Supplement: Supplementary Materials — Figure S1: the workflow of this study. Figure S2: (A) identification of the optimal cluster number by calculating CPI (blue line) and Gaps-statistics (red line) in TCGA-KIRC cohort. (B) Consensus heatmap based on results from 10 multiomics integrative clustering algorithms with the cluster number of 2, 3, 4, and 5, and related quantification of sample similarity using the Silhoutte score based on the consensus ensembles result. Figure S3: heatmap of subtype-specific upregulated and downregulated biomarkers using DEseq2 for the two identified subtypes in TCGA-KIRC cohort. Figure S4: (A) DNA substitution types including transition (Ti) and transversion (Tv) among CS1 and CS2. (B) The most significant difference of mutated genes between CS1 and CS2. (C) The lollipop plot illustrates the differential distribution of variants for PBRM1. (D) Kaplan-Meier curves show the independent relevance between overall survival and PBRM1 mutation in CS1 and CS2 subgroups. (E) The lollipop plot illustrates the differential distribution of variants for PBRM1. (F) Potential druggable gene categories from mutation dataset in CS1 and CS2. Figure S5: (A) differences in oncogenic signaling pathways between the CS1 and CS2 subgroups. (B) Heatmap of oncogenic pathways based on ssGSEA algorithm in the CS1 and CS2 subgroups. Figure S6: heatmap of oncogenic pathways based on the ssGSEA algorithm in the CS1 and CS2 subgroups. Figure S7: (A) heatmap of the immune-related gene family of chemokines and receptors, coinhibitors, costimulators, interferons, and receptors and MHC expression in the CS1 and CS2 subgroups. (B) Expression of immune checkpoints in CS1 and CS2 subgroups and difference in ESTIMATE scores between the subgroups. Figure S8: the structure tomographs of the candidate small-molecule drugs for the CS1 subgroup. Figure S9: (A) heatmap of NTP in outer KIRC cohort using subtype-specific upregulated biomarkers identified from Japan ccRCC cohort. (B) Kaplan-Meier survival curve of the two [file 3617775.f1.zip › FigureS8 (1).pdf]

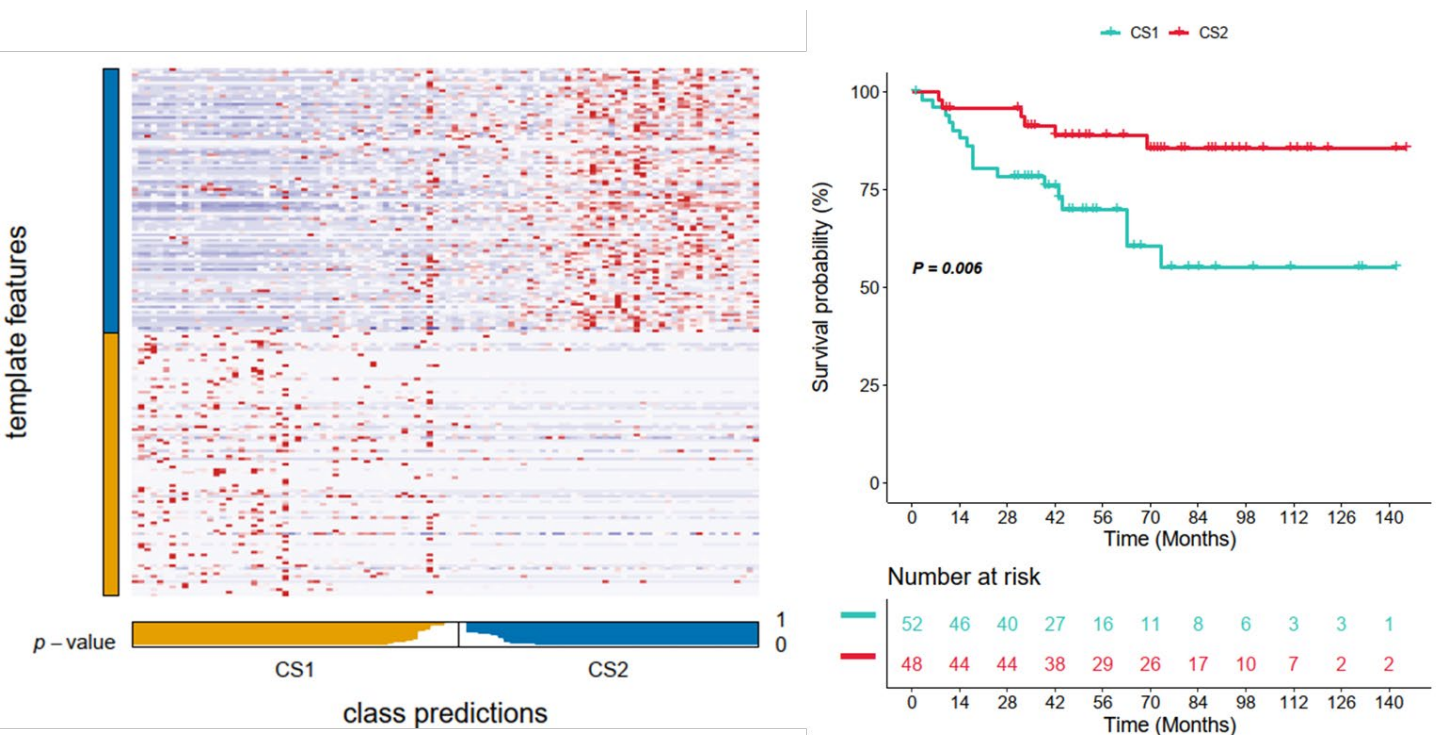

Figure S9

Supplement: Supplementary Materials — Figure S1: the workflow of this study. Figure S2: (A) identification of the optimal cluster number by calculating CPI (blue line) and Gaps-statistics (red line) in TCGA-KIRC cohort. (B) Consensus heatmap based on results from 10 multiomics integrative clustering algorithms with the cluster number of 2, 3, 4, and 5, and related quantification of sample similarity using the Silhoutte score based on the consensus ensembles result. Figure S3: heatmap of subtype-specific upregulated and downregulated biomarkers using DEseq2 for the two identified subtypes in TCGA-KIRC cohort. Figure S4: (A) DNA substitution types including transition (Ti) and transversion (Tv) among CS1 and CS2. (B) The most significant difference of mutated genes between CS1 and CS2. (C) The lollipop plot illustrates the differential distribution of variants for PBRM1. (D) Kaplan-Meier curves show the independent relevance between overall survival and PBRM1 mutation in CS1 and CS2 subgroups. (E) The lollipop plot illustrates the differential distribution of variants for PBRM1. (F) Potential druggable gene categories from mutation dataset in CS1 and CS2. Figure S5: (A) differences in oncogenic signaling pathways between the CS1 and CS2 subgroups. (B) Heatmap of oncogenic pathways based on ssGSEA algorithm in the CS1 and CS2 subgroups. Figure S6: heatmap of oncogenic pathways based on the ssGSEA algorithm in the CS1 and CS2 subgroups. Figure S7: (A) heatmap of the immune-related gene family of chemokines and receptors, coinhibitors, costimulators, interferons, and receptors and MHC expression in the CS1 and CS2 subgroups. (B) Expression of immune checkpoints in CS1 and CS2 subgroups and difference in ESTIMATE scores between the subgroups. Figure S8: the structure tomographs of the candidate small-molecule drugs for the CS1 subgroup. Figure S9: (A) heatmap of NTP in outer KIRC cohort using subtype-specific upregulated biomarkers identified from Japan ccRCC cohort. (B) Kaplan-Meier survival curve of the two [file 3617775.f1.zip › FigureS9 (1).pdf]
